# Synthesis of core–shell structured FAU/SBA-15 composite molecular sieves and their performance in catalytic cracking of polystyrene

**DOI:** 10.1080/14686996.2017.1396561

**Published:** 2017-11-20

**Authors:** Jinlong Du, Chunwei Shi, Wenyuan Wu, Xue Bian, Ping Chen, Qingzhu Cui, Zhixuan Cui

**Affiliations:** ^a^ College of Chemistry, Chemical Engineering and Environmental Engineering, Liaoning Shihua University, Funshun, China; ^b^ Metallurgy Institute, Northeastern University, Shenyang, China; ^c^ Department of Applied Organic Materials Engineering, Inha University, Incheon, Korea

**Keywords:** Core–shell, composite molecular sieve, growth mechanism, FAU, SBA-15, 50 Energy Materials, 103 Composites

## Abstract

Composite molecular sieves, FAU/SBA-15, having core-shell structure were synthesized. The synthesized composite sieves were characterized by X-ray diffractometry (XRD), scanning electron microscopy (SEM), transmission electron microscopy (TEM), energy dispersive X-ray spectroscopy (EDS), pyrolysis fourier transform infrared (Py-FTIR) spectroscopy, temperature programmed desorption spectra (NH_3_-TPD), UV Raman spectroscopy, nuclear magnetic resonance (NMR) and other techniques. XRD, SEM, TEM, N_2_ adsorption-desorption, mass spectrometry, NMR and EDS results showed that the composite molecular sieve contained two pore channels. Py-FTIR results showed that the addition of HY molecular sieves improved the acidity of the composite zeolite. The crystallization mechanism during the growth of FAU/SBA-15 shell was deduced from the influence of crystallization time on the synthesis of FAU/SBA-15 core-shell structured composite molecular sieve. HY dissociated partially in H_2_SO_4_ solution, and consisted of secondary structural units. This framework structure was more stable than its presence in the isolated form on the same ring or in the absence of Al. Thus it played a guiding role and connected with SBA-15 closely through the Si-O bond. This resulted in the gradual covering of the exterior surface of FAU phase by SBA-15 molecular sieves. The presence of SBA-15 restricted the formation of the other high mass components and increased the selectivity towards ethylbenzene.

## Graphical abstract

**Figure:**
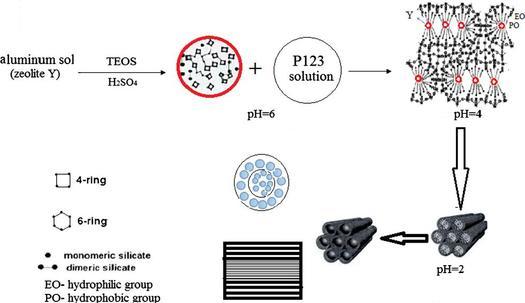

## 1. Introduction

Y-type molecular sieves (FAU type) have unique pore structures and good thermal stabilities [1]. However, the strong acidic nature of Y molecular sieves lowers their performances in shape-selective catalytic reactions. The acidity on the surface and in the pores should be manipulated to enhance their catalytic effects. Ordinary methods of modification not only mask the acidic sites on the surface of the Y-type molecular sieves but also cause acidic sites inside the pores to lose their activity, thereby reducing the catalytic activity [2].

Recent years have seen the emergence of a new type of composite molecular sieves with core–shell structure [3–10]. The advantage is that a pure-silica molecular sieve (e.g. Silicalite-1, SBA-15) is grown on the external surface of an active core (e.g. ZSM-5) [11]. This regulates the acidity on external surface of the molecular sieve and provides entry to large molecules and brings about their initial degradation [12]. The high acidity on the internal surface of the nuclear phase of molecular sieves favors further degradation of the primary products and the diffusion of the final products (relatively small molecules) into the smaller pores of the nuclear phase. This prevents blocking of the pores and thus solves problems [13], such as easy deactivation of catalysts and low yields of high value-added liquid products (too many gaseous products), etc., compared to traditional microporous catalysts used in the catalytic cracking of macromolecules.

Some methods can be used to synthesize core–shell composite molecular sieves [14,15]. Compared to the isomorphous method, the epitaxial method involves a phase comprising of grains on the surface [16], and results in the formation of composite molecular sieve catalyst with core–shell structure. EMT/FAU composite core–shell molecular sieves were successfully synthesized by Goosens et al. [17]. In this synthesis, the zeolite films having FAU structure were oriented on compositionally different micro-sized EMT zeolite crystals. In this synthesis, the zeolite films having FAU structure were oriented on compositionally different micro-sized EMT zeolite crystals.

Composite molecular sieves with core–shell microporous and mesoporous structure [18–20] showed good conversion and selectivity for the catalytic degradation reactions of some macromolecules Zhou et al. [21,22] synthesized c-TUD-1c-Ti-TUD-1 using dry gel conversion method, in which the porous structure of the composites showed better catalytic performance in organic reactions involving macromolecules containing benzene rings compared to traditional zeolites, in addition to good reusability of the catalysts. The cracking of the benzene ring-containing macromolecules, such as 1,3,5-tripropyl benzene does not require the catalyst to be highly acidic, but instead must possess good porosity. The composite porous core–shell material ZSM-5 obtained by base treatment [23], showed relatively high conversion and good stability in the catalytic cracking of 1,3,5-tripropyl benzene. Zhao’s research group prepared ZSM-5@β core–shell molecular sieves [24], in which the porosities and the multilevel acid centers of the composites were beneficial for improving the yield of propylene in the catalytic cracking of macromolecular byproducts (C4-C10 hydrocarbons) [25].

Detailed study on the synthesis of core–shell composite molecular sieves using the microporous molecular sieves Y, with high acidity and high catalytic activity, equivalent to that of ZSM, has been rarely reported. Therefore, in this work, a secondary method has been employed to synthesize FAU/SBA-15 type core–shell composite molecular sieves, to extend the types of core–shell composite molecular sieves and their applications in the macromolecular catalysis. The crystallization mechanism of the shell growth in Y/SBA-15 was deduced, based on the characterization results. Moreover, its effect on the catalytic cracking of polystyrene has also been investigated.

## 2. Material and methods

### 2.1. Reagents and raw materials

Sulfuric acid (H_2_SO_4_), sodium silicate (water glass), and tetraethyl orthosilicate (TEOS) (chemically pure) were procured from Shenyang Chemical Reagent Factory. (PEO20-PPO70-PEO20) block copolymer (P123) was obtained from Sigma-Aldrich Shanghai. *N*-hexane (analytically pure) was purchased from Shenyang Chemical Reagent Factory, whereas polystyrene (PS) particles were purchased from Fushun Catalyst Factory.

## 2.2. Experimental methods

### 2.2.1. Synthesis of Y molecular sieves

Mix some amount of sodium aluminate, sodium hydroxide, and deionized water in a 100 ml beaker, at 5–20 °C until complete dissolution. Slowly add the sodium silicate (water glass) solution into the fully dissolved sodium aluminate solution, and make the molar composition of the whole: 16 Na_2_O:Al_2_O_3_:15 SiO_2_:320 H_2_O. After forming the seed solution, it was stirred for 30 min and aged at room temperature for 2–4 days.

To make alumino silica gel, mix a certain amount of sodium aluminate, sodium hydroxide, and deionized water in a 500 ml beaker, and stir until complete dissolution. Add aged seed solution at room temperature, and make the final molar composition of the reactants: 4.3 Na_2_O:Al_2_O_3_:10 SiO_2_:180 H_2_O. After some further stirring, crystallize the mixture in stainless steel crystallization kettle at 90 °C. Filter and wash at 110 °C, and the NaY-type molecular sieves were obtained after drying. The ratio of Si to Al of NaY was 2.44, which was determined by X-ray fluorescence spectroscopy (XRF) [26].

The NH_4_Cl solution (0.5 mol/L) was exchanged with 10 g NaY zeolite for 24 h. The exchanged NaY zeolite was dried in an oven at 110 °C for 12 h, and then calcined in a muffle furnace at 450 °C for 6 h to obtain HY. The reason for using HY instead of NaY is that the activity of HY is higher than that of NaY, which is favorable for the degradation of polystyrene.

### 2.2.2. Synthesis of core–shell FAU/SBA-15 composite molecular sieves

The tri-block copolymer P123, H_2_O, and TEOS were mixed together in a three-necked flask and 10 mol/L sulfuric acid (H_2_SO_4_) was dropped slowly to adjust the pH values to 7, 6, 4, 2, or 1. Meanwhile small amounts of Y crystals were added multiple times. The mass ratios of reactants were: *m*(P123): *m*(H_2_O): *m*(TEOS): *m*(HY) = 4:30:9.5:4. The mixture was aged at 38 °C for 12 h and crystallized in an oven at 100 °C for 24 h. The hydrothermally synthesized sample was obtained after centrifugation, washing, drying, and calcination at 550 °C. The prepared core–shell material was denoted as SYS_*p*_, where *p* represented the pH of the solution, and *p* = 7, 6, 4, 2, or 1.

### 2.3. Characterization methods

X-ray diffraction (XRD) analysis of the powdered samples were conducted using an X-ray diffractometer (XRD-6000, Shimadzu, Japan) using CuK*α*1 source, with tube voltage of 40 kV and tube current of 80 mA. The step length in small-angle XRD (SXRD) tests was 0.01°, with a scanning rate of 2°/min in the range of 0.4–0.8°. The step length in wide-angle XRD (WXRD) tests was 0.1°, with scanning rate of 8°/min in the range of 10–70°. Surface morphology of the samples was studied by scanning electron microscopy (SEM) at a magnification of 250,000–1,000,000 microscope (SEM) with a magnification of 250,000–1,000,000 X at an accelerating voltage of 0.1–30 kV. The N_2_ adsorption–desorption isotherms were measured at –196 °C with an Auto Chemistry 2010 instrument. Transmission electron microscopy (TEM) images were obtained using a JEM-2100 TEM from JEOL (Japan) that operated at an accelerating voltage of 200 kV.

The quality scanning range of a LTQ XL linear ion mass spectrometer (Thermo, USA) in negative ion detection mode, equipped with ESI electrospray ion source, is *m*/*z* 100–900 Da, spray voltage 4 kV, and capillary voltage –20 V.

Measured by a Bruker MSL-300 nuclear magnetic resonance spectrometer (Germany), the resonant frequency of ^27^Al magic-angle spinning nuclear magnetic resonance (MAS NMR) is 78.205 MHz, with the rotor rotating frequency 3 kHz, the RF field frequency 51 kHz, the reverse angle 18°, and the cycle time 500s; the resonant frequency of ^29^Si MAS NMR is 59.592 MHz, the rotor rotation frequency 4 kHz, the RF field frequency 37 kHz, the reverse angle 60°, and the cycle time 2s.

Pyrolysis Fourier transform infrared (Py-FTIR) spectra were recorded on a Bio-Rad FTS15/C spectrometer (Shimadzu, Japan) to determine the surface acid site types (B acid, L acid). UV Raman spectroscopy was carried out on a RM2000 Raman spectrometer (Renishaw, UK). Excitation was provided by a helium–cadmium laser at 325 nm and the laser power at the sample position was about 4.5 mW.

### 2.4. Catalytic cracking of polystyrene

A certain amount of polystyrene (PS) particulate sample was placed in the homemade hydrocracking micro-reactor device and a certain amount of catalyst (*m*(SYS_2_):*m*(PS) = 1:2) was added to it and mixed homogeneously. Then the device was made airtight. The device was slowly purged with N_2_ for 5 min and heated up to the decomposition temperature at a heating rate of 20 °C/min for the catalytic degradation to occur. The final temperature for catalyst degradation was 650 °C. The liquid product obtained during degradation was analyzed by liquid chromatography, after condensation.

## 3. Results and discussions

### 3.1. Influence of pH on the formation of core–shell composite molecular sieves

#### 3.1.1. XRD and SEM analysis

SYS_6_, SYS_4_, SYS_2_, and SYS_1_ samples were filtered, dried (not calcined), and then characterized by XRD and SEM to analyze the changes in morphologies and structures of the products in solutions.

**Figure 1.**
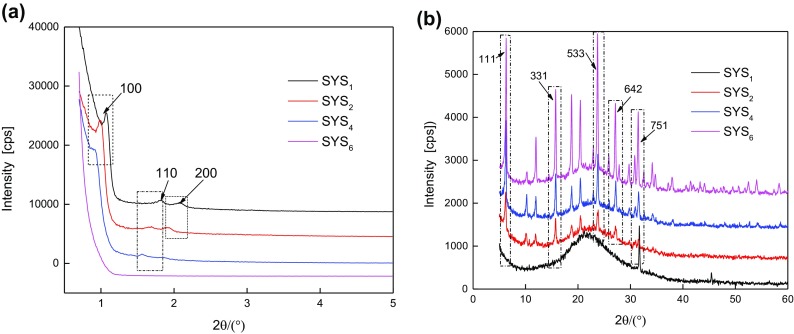
XRD patterns of composite molecular sieves synthesized under different pH conditions: (a) SXRD and (b) WXRD patterns.

**Figure 2.**
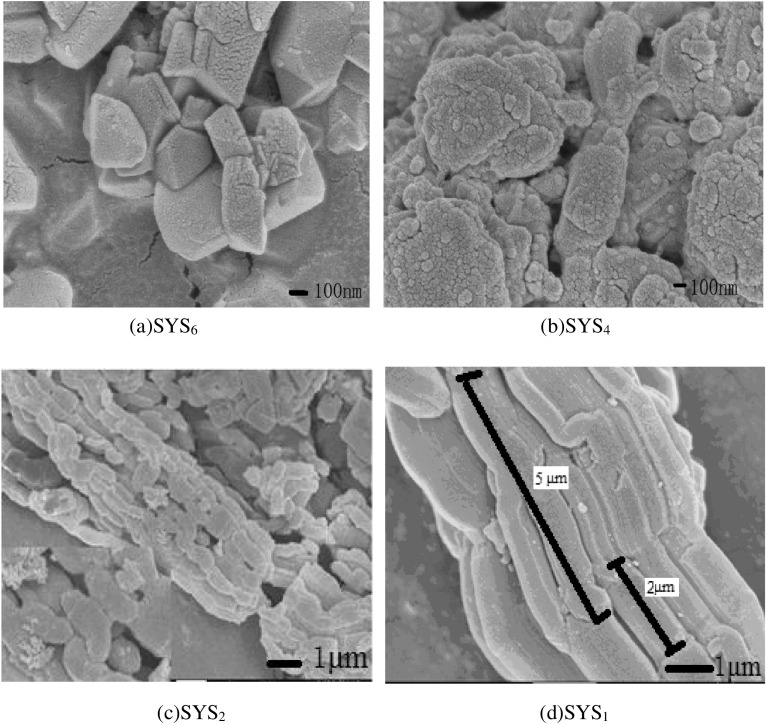
SEM images of SYS composites synthesized under different pH conditions.

Figures 1 and 2 show the XRD patterns and SEM images, respectively, of the continuous tubular core–shell composite molecular sieves during synthesis under different pH conditions. It can be seen from Figures 1 and 2 that with decrease in pH, the crystalline phase underwent gradual compositional changes, until the core–shell structure of the molecular sieve was formed. In case of SYS_6_ (Figure 2(a)), octagon-like and spherical-like agglomerations, with diameters more than 200 nm, were seen in the SEM images. No characteristic peaks of (1 0 0), (1 1 0), or (2 0 0) crystal planes appeared, suggesting that this sample did not possess a typical mesoporous structure. Very weak peaks appeared in the wide-angle region, which were characteristic of a microporous structure. These were probably due to the primary tetra-ring structural units formed due to the corrosion of Y-type microporous molecular sieves by H_2_SO_4_ and the disappearance of some of the characteristic surface microporous structures. In case of SYS_4_ sample (Figure 2(b)), multiple octagon-like and sphere-like agglomerations were present, which showed more rod-like structures compared to the SYS_6_ sample. By combining this result with the SYS_4_ pattern in Figure 1, it was evident that weak characteristic peaks of (1 1 0) and (2 0 0) crystal planes appeared in the small-angle region, which indicated that the characteristic mesoporous structure became more obvious. The SXRD of SYS_4_ showed some characteristic peaks of microporous structure, but their intensities were low. Hence, it could be deduced that Y was coated with some amount of SBA-15 and was protected from corrosion from H_2_SO_4_. Hence, some peaks characteristic of microporous structure could be seen. In case of SYS_2_ sample (Figure 2(c)), the magnified image at the lower left corner showed continuous tubular morphology. Combining the morphology of SYS_1_ sample in Figure 2(d) with the SYS_1_ pattern in Figure 1(b), it was evident that due to highly acidic environment, the Y-type zeolite was corroded completely and showed only the mesoporous form of the composite.

As the pH was lowered, the acid not only provided the required H^+^ ions for the formation of mesopores, but also partially removed Al from the Y-type zeolite framework. When the Y-type microporous molecular sieve was mixed with P123 solution and the pH value was adjusted from 6 to 2, the original microporous structure was coated with mesoporous molecular sieve. The adjustment of pH had influence on two aspects of the synthesis of the core–shell structure of the molecular sieve products. The first aspect was promotion of agglomeration of amorphous silicon and aluminum species in the system and the subsequent formation of mesoporous phase structure. The second aspect was breaking of the chemical equilibrium between the amorphous silicon and aluminum species. The Y-type microporous zeolite crystals in the system resulted in the partial destruction of the zeolite structure. This destruction did not degrade the structure completely to the amorphous Si–O or Al–O tetrahedral structure, but instead degraded it to the microporous structural units.

#### 3.1.2. Changes in silicon species

With lowering of pH, the silicon species in the mesoporous system mainly underwent the following two reaction steps.

The first step was carried out under weakly acidic conditions:

$${\text{SiO}}_{3}^{2-}+2{\text{H}}^{+}={\text{H}}_{2}{\text{SiO}}_{3}$$

**Figure 3.**
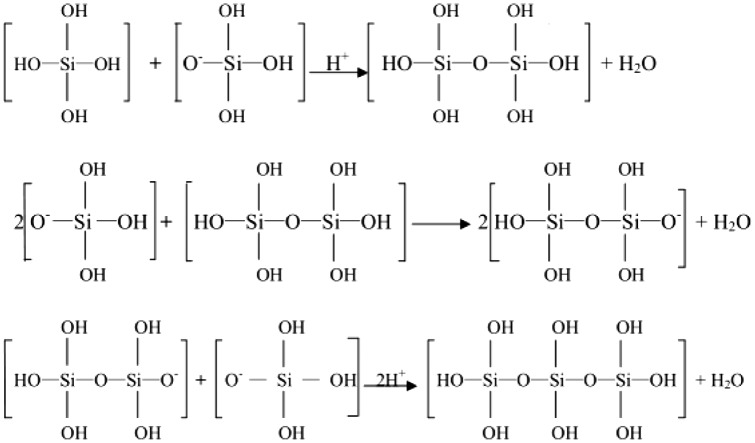
The second step of silicon species in the mesoporous system mainly underwent.

The second step was carried out by gradually increasing the acidity (Figure 3):

It could be seen that the introduction of acid into the weakly acidic medium, provided H^+^ continuously (pH = 6). This shifted the chemical equilibrium of the reaction in the first step to the right side. This reversible reaction medium showed some buffer-like properties in maintaining the pH value and had protective effect on the Y-type microporous zeolite structure. As the acidity increased gradually (<pH 6 < 2), a rapid polymerization of the silicates occurred in the second step, which promoted the formation of the mesoporous phase (pH = 2). Increase in the amount of acid (pH = 1) introduced led to an increase in amount of aluminum removed from the framework and consequently the degradation of FAU type crystals.

Hence, the structure of the synthesized FAU/SBA-15 composite molecular sieves was dependent on pH. Decrease in pH during the synthesis caused the destruction of the crystalline structure of FAU, and meanwhile this structure was coated with the newly formed SBA-15. Thus, the core–shell structure of the composite molecular sieve with FAU as core and SBA-15 as shell was obtained with both, microporous and mesoporous structures.

### 3.2. Characterization of the core–shell structure of composite molecular sieves

#### 3.2.1. TEM

**Figure 4.**
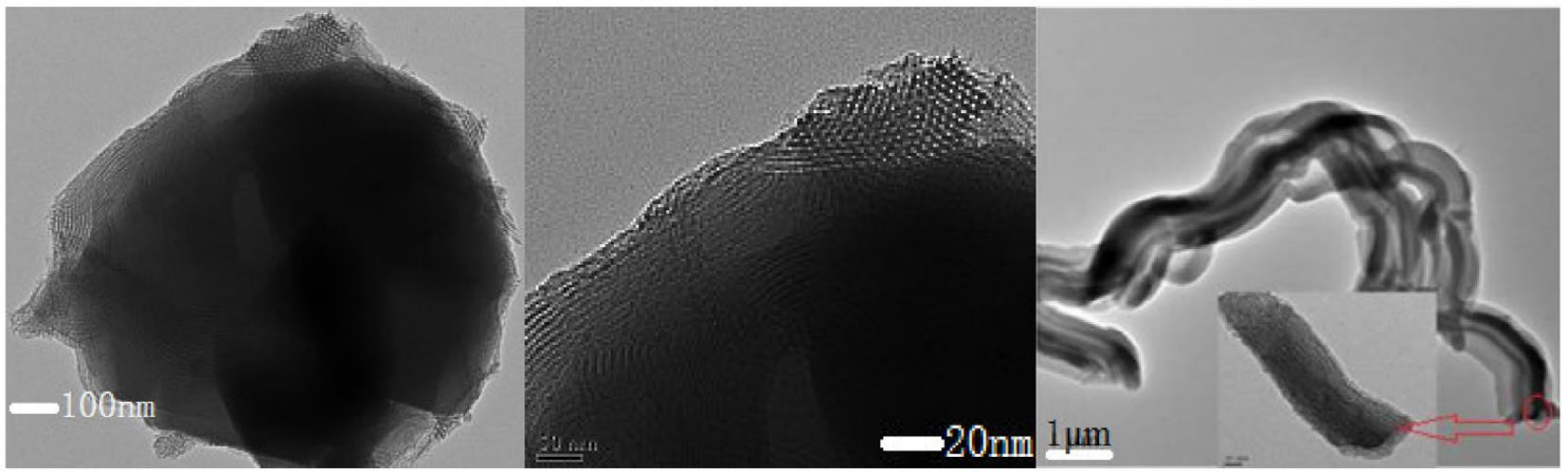
TEM images of core–shell structure SYS_2_: the boundary between micropores and mesopores (a), cross section (b), overall morphology (c).

It could be further seen from TEM images (Figure 4) that the shell phase SBA-15 coated on the external surface of the core phase FAU was highly dense with no cavities or vacancy defects (Figure 4(b)). The boundary between micropores and mesopores could be clearly seen (Figure 4(a)) and the whole shape was continuously tubular (Figure 4(c)). The middle region was darker. The structure with smaller pore size was microporous (core phase) while the exterior structure with a larger pore size was the mesopore (the thickness of shell phase was 100–200 nm).

**Figure 5.**
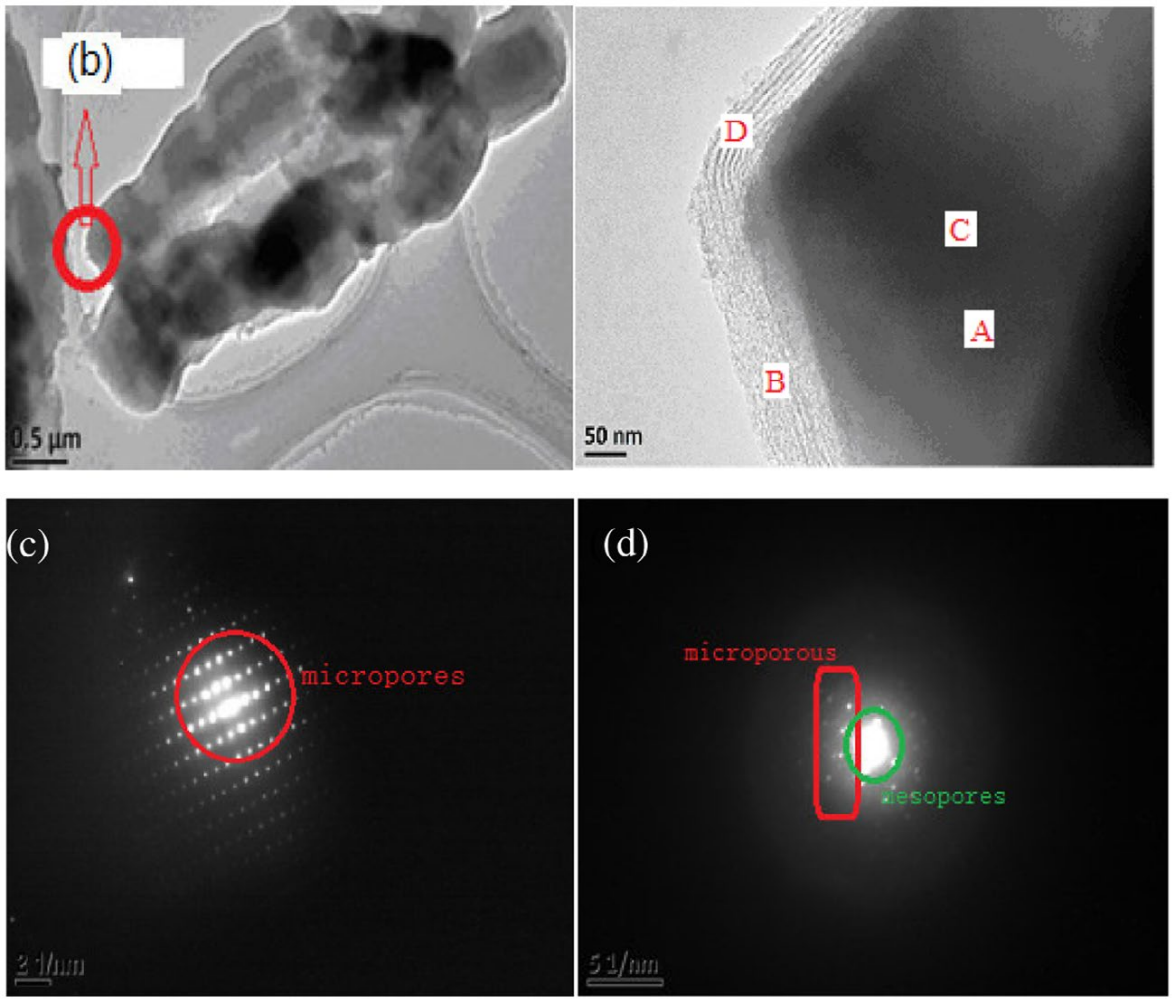
The overall morphology of core–shell composite sieves (a), enlarged image of a section of the core–shell composite sieve (b), diffraction patterns of Y molecular sieves (c), and diffraction patterns of core–shell composite molecular sieves (d).

Figure 5 further demonstrated the coexistence of microporous and mesoporous structures in the core–shell composite molecular sieves (sample SYS_2_).

Figure 5(a) shows the overall surface topography of the sample (SYS_2_). Figure 4(b) shows the magnified image of a section of Figure 5(a), in which the boundary between the core microporous and shell mesoporous phases was clear. Figure 5(c) shows the diffraction pattern of Y-type microporous molecular sieves, which has uniform spots. Figure 5(d) shows the coexistence of large spots and small spots in the diffraction patterns of the composite molecular sieve, indicating that the core–shell composite molecular sieve contained two pore channels of micropores and mesopores, instead of the microporous molecular sieves with single pore size.

#### 3.2.2. Elemental analysis

In order to further prove the core–shell structure of SYS_2_, energy-dispersive spectroscopy (EDS) measurements were performed at four points, sequentially from the interior to the exterior, as shown in Figure 5(b) and the analytic results are presented in Table 1.

**Table 1.** Elemental analysis of SYS_2_ by TEM/EDS.

Point	Weight percentages of elements				Atomic percentages of elements			
	O	Na	Al	Si	O	Na	Al	Si
A	0.506	0.016	0.028	0.449	0.641	0.014	0.058	0.288
B	0.532	0.007	0.016	0.445	0.665	0.006	0.004	0.325
C	0.493	0.017	0.024	0.451	0.639	0.015	0.062	0.285
D	0.539	0.007	0.017	0.442	0.673	0.005	0.004	0.318

Elements such as Si, Al, O, and Na were found to be present in the composite molecular sieve. This was consistent with the different elements present in the raw materials, used for synthesizing the molecular sieves. Moreover, their intensities were also in accordance with the amounts of the raw materials added. More specifically, the atomic ratios of Si:Al at A and C points were 5 and 4.6 (close to the atomic ratios of Si:Al in Y molecular sieves), and the atomic ratios of Si:Al at B and D points were 81.25 and 91 (close to the atomic ratios of Si:Al in SBA-15 molecular sieves). This was a further indication that the composite molecular sieve had a core–shell structure.

#### 3.2.3. Porosity analysis

**Figure 6.**
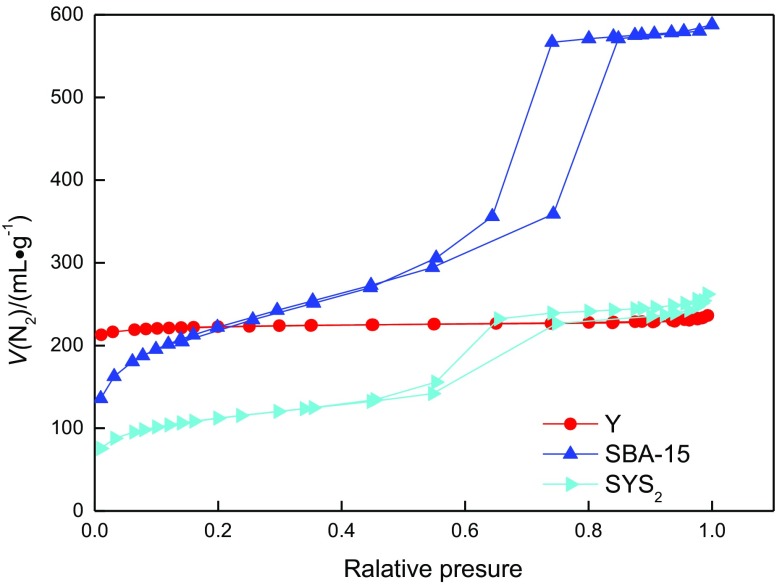
N_2_ adsorption–desorption isotherms of Y, SBA-15, and SYS_2_.

Analysis of N_2_ adsorption desorption Y, SBA-15, SYS_2_ adsorption desorption isotherms is shown in Figure 6.

It can be seen from Figure 6, the Y adsorption desorption isotherms for I, microporous molecular sieve is a typical line; adsorption–desorption isotherms for SBA-15 type V is mesoporous molecular sieve type typical line, and SYS_2_ IV line is typical at low relative pressure stage. The N_2_ adsorption–desorption isotherms of SYS_2_ composite molecular sieves showed obvious microporous and mesoporous characteristics, which demonstrated that it was composite molecular sieves with composite pore structures. The detailed properties are given in Table 2.

**Table 2.** Surface area and average desorption pore volume of SYS_2_ sample. BET stands for Brunauer–Emmett–Teller and BJH for Barrett–Joyner–Halenda.

Sample	$S_{\text{BET}}$/(m^2^·g^−1^)	*V*_BJHDesorption_/(cm^3^·g^−1^)
SYS_2_ (mesoporous)	406.588	0.326
SYS_2_ (micropores)	493.149	0.247

**Figure 7.**
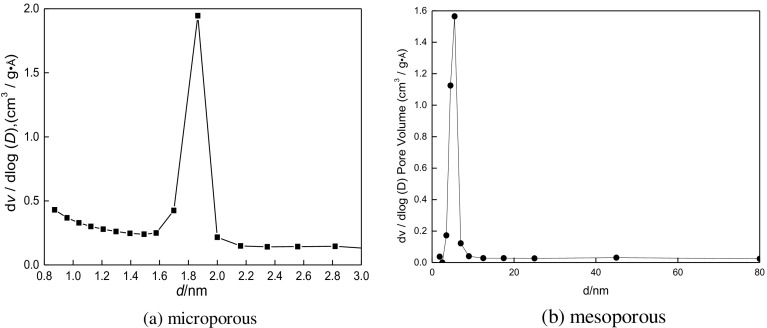
The pore size distribution curve of the micro- and mesoporous of SYS_2_.

The distribution of pore sizes is shown in Figure 7. The sizes of micropores and mesopores are approximately 1.85 and 6 nm, respectively.

### 3.3. Analysis of acid sites

**Figure 8.**
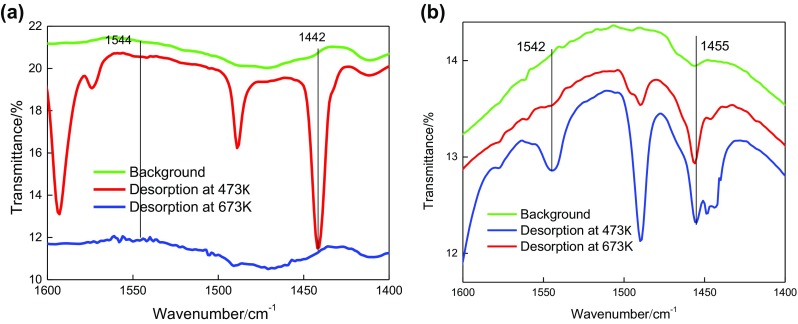
Py-FTIR spectra of pyridine desorbed on (a) NaY/SBA-15 and (b) HY/SBA-15.

Pyridine is absorbed on the acid sites of the composites (HY/SBA-15 and NaY/SBA-15). The peaks at 1542 and 1450 cm^−1^, due to chemisorbed pyridine, are used to determine the number of acid sites on the composite. The ratio of the peak (1442 cm^−1^) was weak that indicates that both Na^+^ and non-framework aluminum species in NaY/SBA-15 zeolite are weak L acid sites. The peaks at 1450 and 1542 cm^–1^ can be attributed to the C–C stretching vibrations of the pyridine complex with the Lewis acid and that of the pyridine complex with the Brønsted acid, respectively. The Brønsted acid of HY/SBA-15 is stronger than NaY/SBA-15.The infrared spectrum of pyridine showed that the addition of HY improved the acidity of the composite zeolite. The results of Py-FTIR showed that the addition of HY improved the acidity of the composite zeolite (see Figure 8).

The results of acid content and distribution of HY, NaY/SBA-15, and HY/SBA-15 molecular sieves obtained from the temperature-programmed desorption (NH_3_-TPD) spectra [26] are shown in Table 3. Compared to NaY/SBA-15, the proportion of medium and strong acids of HY/SBA-15 increased, while the proportion of weak acids decreased.

**Table 3.** The acidity and acidic distribution of three kinds of molecular sieves.

Samples	Distribution of acid strength/%			Total amount of acid/(mmol·g^−1^)
	Strong	Medium	Weak	
HY	27.9	33.2	38.9	1.70
NaY/SBA-15	24.60	38.79	37.61	0.738
HY/SBA-15	20.87	42.95	36.18	0.823

## 4. Mechanism of crystallization

### 4.1. UV Raman spectroscopic analysis

UV Raman spectroscopy can prevent disturbances due to fluorescence, generated by the intermediate species during the synthesis of molecular sieves and enhance the sensitivity of the measurement. Meanwhile, this characterization method showed much higher recognition sensitivity toward templates than XRD, which provided the possibility of studying the *in situ* mechanism of synthesis of molecular sieves using *in situ* UV Raman spectroscopy [27–29].

**Figure 9.**
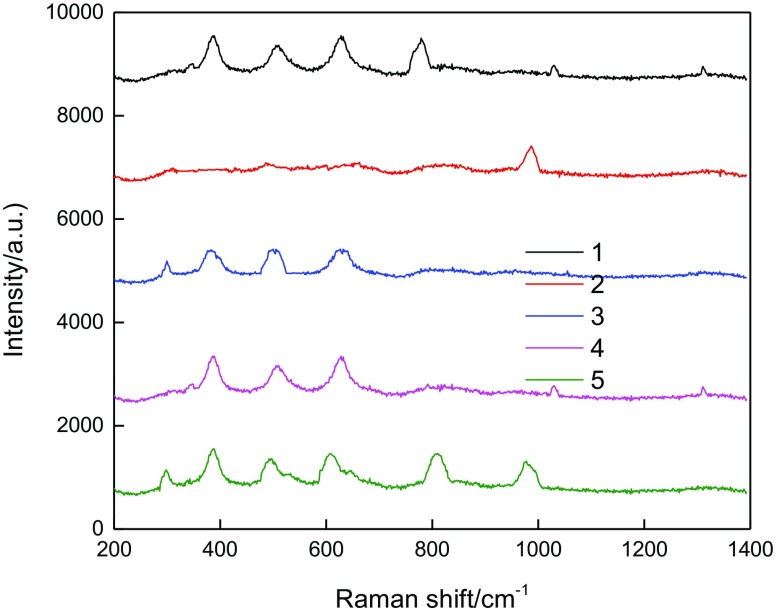
*In situ* UV Raman spectra of liquid and solid phases in the crystallization.

Figure 9 shows the UV Raman spectrograms of the liquid phase and solid phase during the crystallization process of the composite molecular sieves.

Characteristic bands at 380, 520, and 610 cm^−1^, attributed to P123, appeared in all the spectra except for spectrum 2. During the crystallization process of the core–shell of the composite molecular sieve, the template agent P123, which was initially in both the solid and liquid phases, was transformed completely to the solid phase. This indicated that when the pH of the system was reduced, the silicon species (positive charges) agglomerated, due to electrostatic interactions, around the P123 micelles (negative charges) in the liquid phase. This transformed the precipitates formed, by coating P123 in the liquid phase onto the exterior surface of Y to form the solid gel phase. The formed mesoporous phase showed a highly ordered two-dimensional hexagonal structure (the morphologies of the samples after crystallization in hot water can be seen in Figure 4).

Compared to spectrum 4, spectrum 3 showed a new characteristic peak at 298 cm^−1^ and the initial peak at 520 cm^−1^ shifted to 514 cm^−1^, both indicating the long-range ordered environment of the structural unit of HY [30]. In spectrum 1, the band at 778 cm^−1^ could be assigned to the oligomeric silicon species in the liquid phase [31]. HY dissociated partially in H_2_SO_4_ solution and formed the secondary structural unit. The characteristic singlets at 1024 and 1310 cm^−1^ were attributed to the silicon species in the liquid phase. When the pH was reduced to 2, the gelation increased markedly and the system became very viscous. In spectrum 2, only one band at 985 cm^−1^, attributed to ${\text{SO}}_{4}^{2-}$, could be observed. The appearance of this band was due to the addition of H_2_SO_4_ to adjust the pH of the system [32]. In spectrum 5, the characteristic bands at 298 and 380 cm^−1^ were assigned to the breathing vibrations of dual-hexatomic rings, whereas the typical vibrational bands of SBA-15 appeared at around 500, 606, 808, and 976 cm^−1^. Among these, the broad band at around 500 cm^−1^ overlay the vibrational bands of the four-, five-, and six-membered rings in the mesoporous molecular sieves. The band at 606 cm^−1^ was probably due to the Si–O bridged bond in the mesoporous molecular sieve. The band at 808 cm^−1^ could be attributed to the Si–O–Si symmetric stretching vibration in the molecular sieve, and the band at 976 cm^−1^ was a result of the surface Si–OH groups [33,34].

### 4.2. Possible mechanism of the formation of FAU/SBA-15 composite core–shell structured molecular sieve

**Figure 10.**
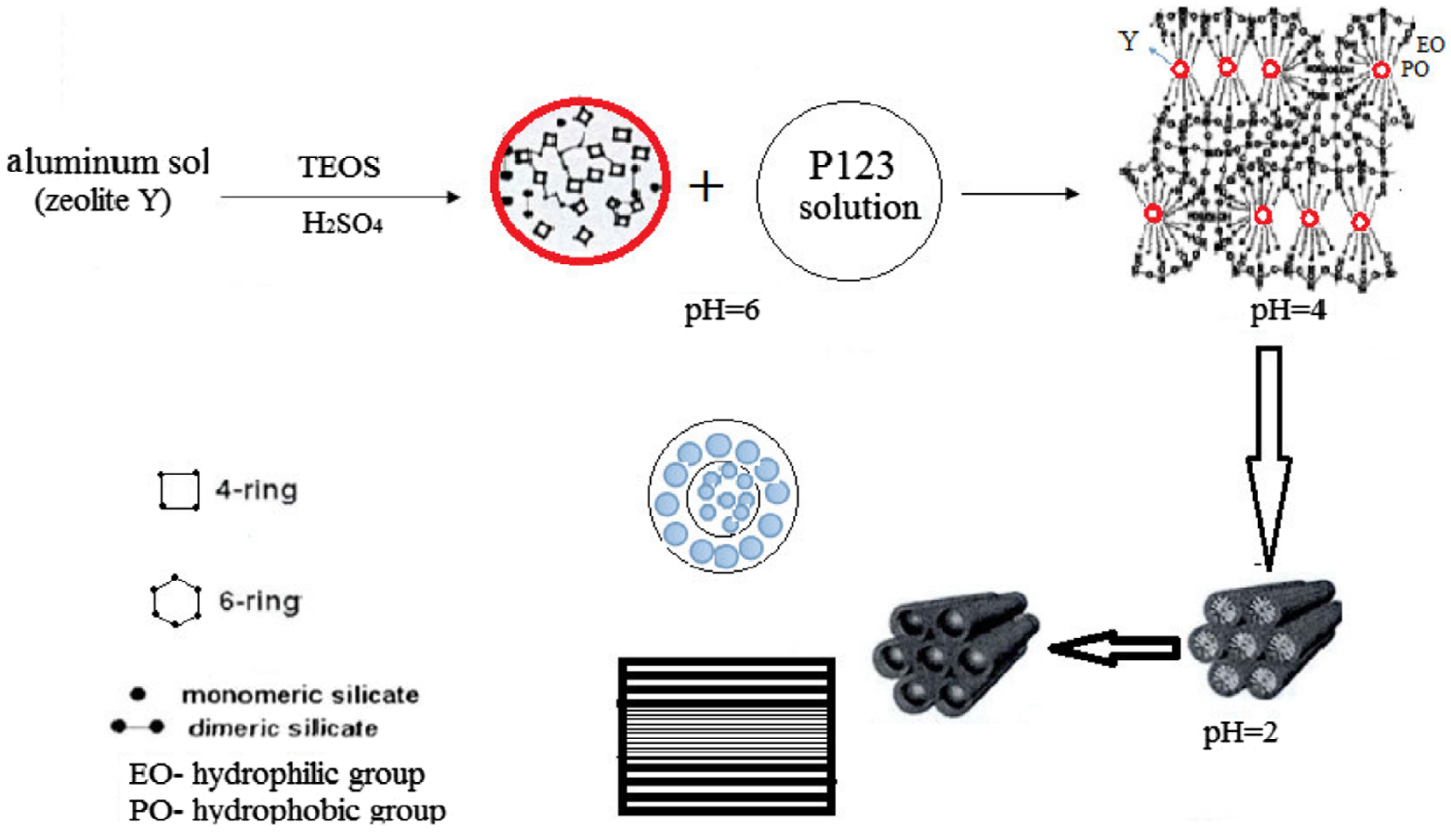
Scheme showing the synthesis of FAU/SBA-15 core–shell composite.

From the above results, the mechanism for the formation of the FAU/SBA-15 core–shell structured molecular sieve was proposed (Figure 10).

After the addition of octagonal HY crystal particles into the SBA-15 synthetic crystallization environment, the gelation around the Y crystals (FAU phase) changed with variation of pH. HY dissociated partially in the H_2_SO_4_ solution (pH = 6) and consisted of secondary structural units.

**Figure 11.**
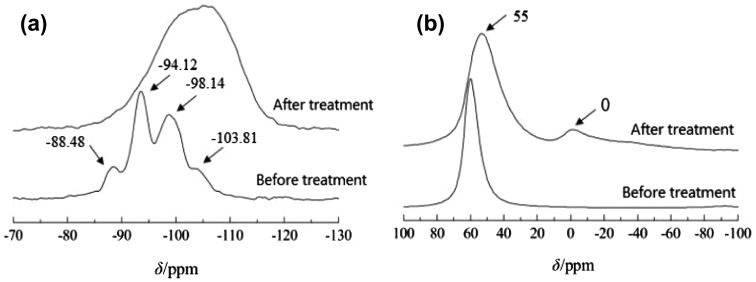
^29^Si(a) and ^27^Al(b) MAS NMR spectra of H_2_SO_4_-treated Y zeolite samples.

The result of MAS NMR and Mass spectrometry are supportive in the analyses secondary structural units (see Figure 11).

The peaks in the ^29^Si spectra of HY molecular sieves when *δ* = –88.48, –94.12, –98.14, and –103.81 are in turn attributed to Si (3Al), Si (2Al), Si (1Al), and Si (0Al) in their skeletons. The facts that the signals move toward high field and are obviously broadened after acid treatment indicate that the zeolite skeleton has been destroyed and become amorphous, producing a large amount of Si–OH. Meanwhile, the proportion of Si species with low Al content is increased, which indicates that Al is removed from the sample after acid treatment.

From the ^27^Al MAS NMR spectra, the acid treated HY molecular sieve appears a new peak at *δ* = 0, which shows that acid treatment turns the original HY molecular sieve with six-coordinated Al on the skeleton part to four-coordinated Al. It can be seen that the peak of the four-coordinated Al at *δ* = 55 is obviously broadened, which indicates that the environment uniformity of the four-coordinated Al has been improved, and the secondary structure unit with tetra-atomic ring structure has been obtained [35].

**Figure 12.**
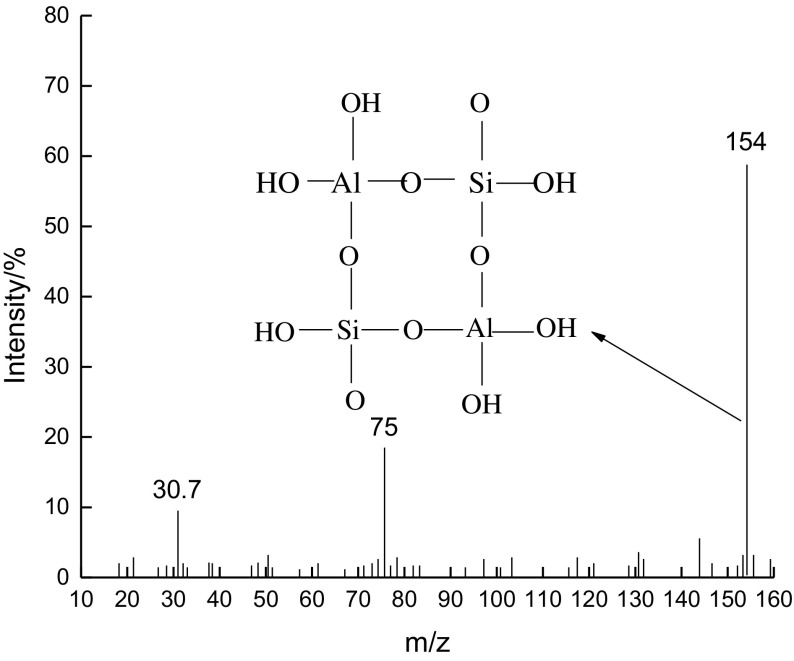
Mass spectra of secondary structural units.

Using electrospray ionization mass spectrometry, we studied the secondary structural units in which HY is dissolved in the liquid phase of H_2_SO_4_, as is shown in Figure 12.

As can be seen from Figure 12, the mass spectra values *m*/*z* = 30.7 and 75 correspond to C_6_H_3_ and ${\text{HSiAlO}}_{3}^{3+}$, respectively. The major fragmentation peak of the secondary structure is ${\text{H}}_{6}{\text{Al}}_{2}{\text{Si}}_{2}{\text{O}}_{12}^{2-}$ (*m*/*z* = 154), and we have speculated about the structure of the tetra-atomic ring.

It was proved by the self-consistent field molecular orbital calculation that, considering the energy aspect, Al preferably existed on the tetra-rings. This framework structure was more stable than its presence in the isolated form on the same ring or in the absence of Al [36]. Since the bond length of Si–O (0.165 nm) was smaller than that of Al–O (0.175 nm), substitution of Si at the Al position led to irregular contraction of crystal cell and decrease in stability (pH = 4). Thus, it played a guiding role and connected with SBA-15 closely through the Si–O bond (pH = 2). Finally, this resulted in the gradual covering of the exterior surface of FAU phase by SBA-15 molecular sieves [37,38].

## 5. Catalytic degradation of polystyrene by composite molecular sieve

The synthesized SYS_2_ core–shell structured molecular sieves were applied to the catalytic cracking reaction of PS and the analysis of the liquid products is presented in Table 4.

**Table 4.** Reaction products of catalytic degradation and thermal cracking of PS.

Component	*w*(component)/%		
	PS	PS+HY	PS+SYS_2_
Benzene		49.22	58.43
Toluene	1.82	9.86	16.42
Ethylbenzene	3.10	6.46	10.17
Styrene	69.15	4.03	4.36
Cumene		1.47	2.23
Other recombinant components	22.76	15.77	2.39
The amount of coke	3.15	13.18	5.97

The thermal cracking of PS alone produced styrene monomer as the major product. After the introduction of catalyst, the selectivity for benzene, toluene, ethylbenzene, isopropylbenzene, etc. (all belonged to harmonic composition of gasoline with high octane number) increased, which was due to the attack of the proton at Brønsted acid site of the acidic catalyst surface, on the phenyl group of polystyrene. This led to the intermolecular or intramolecular hydrogen transfer, decrease in *β*-type chain scissions, increase in other modes of chain scission, transformation of more of PS to ethylbenzene, and so on. The selectivity toward ethylbenzene increased remarkably when catalyzed with the core–shell structured SYS_2_ molecular sieve, which increased from 6.46% by catalyzed HY with the non-core–shell structured (3.10% by thermal cracking) to 10.17%. Since the interior of the SYS_2_ core–shell structured molecular sieve still retained the Y strong acidic center, and the shell was completely covered with SBA-15, which was weakly acidic, side reaction such as isomerization on the exterior surface was prevented and the conversion of olefin and selectivity to arene showed improvement. Meanwhile, PS degraded initially when passing through the mesopores and the degradation products entering the micropores were smaller. The relatively strong acidic centers in the micropores facilitated the further degradation of the initial products. As a result, the yields of products with smaller molecular weights (e.g. benzene, toluene, ethylbenzene), when catalyzed by SYS_2_, were higher than that catalyzed by HY. Moreover, the yields of higher mass components were also lower than HY. The shell part of the SYS_2_ sample (SBA-15) covered almost the entire exterior surface of FAU forming core–shell structured system with connected pore channels, and enhanced the synergistic catalysis effect. In addition, the existence of the SBA-15 shell restricted the formation of other high mass components and increased the selectivity toward ethylbenzene.

## 6. Conclusions

FAU/SBA-15 core–shell structured composite molecular sieves with two kinds of porous structures, unique selectivity, and active sites were synthesized. The further growth of the molecular sieve shell by crystallization was precisely controlled. The key to the synthesis was to use rational acidity to control the growth of the shell mesoporous phase crystals. The composite molecular sieve possessed two kinds of porous structures and exhibited unique selectivity and activity. During the catalytic cracking of polystyrene, the macromolecular products could pass through the pore channels connecting SBA-15 shell and HY core and were transferred to the active catalytic centers for the reaction. Then, the secondary cracking reaction took place and the molecules were transformed into arenes. Moreover, synergistic effects were shown by the two kinds of molecular sieves. Therefore, the core–shell synthesized composite porous structures showed improved the catalytic cracking of polystyrene, to obtain the harmonic components of gasoline with high octane number, such as ethylbenzene.

## Disclosure statement

No potential conflict of interest was reported by the authors.

## Funding

A part of this work was supported by the Research Fund for the Foundation of Liaoning Education Committee [L2017LQN002]; the Doctoral Program of Higher Education by Chinese Education Department [grant number 20100042110008]; Liaoning BaiQianWan Talents Program, and the Talent Scientific Research Fund of LSHU [grant number 2016XJJ-064].
